# Postmortem and Antemortem Forensic Assessment of Pediatric Fracture Healing from Radiographs and Machine Learning Classification

**DOI:** 10.3390/biology11050749

**Published:** 2022-05-13

**Authors:** Kelsey M. Kyllonen, Keith L. Monson, Michael A. Smith

**Affiliations:** FBI Laboratory, Quantico, VA 22135, USA; kelseymkyllonen@gmail.com (K.M.K.); masmith3@fbi.gov (M.A.S.)

**Keywords:** forensic anthropology, children, fracture dating, healing stage, radiographs, machine learning

## Abstract

**Simple Summary:**

Being able to estimate from X-rays alone how long ago a child’s bone was fractured is important for prosecuting suspected child abuse of living or dead children. This estimate can also help identify a child when all that remains are bones. Experts use various indicators to make these estimates of the age of healing and fully healed fractures, in living and deceased persons, even years after the injury occurred. However, it is not a precise science. We proposed a method using a new combination of indicators to classify fracture healing in children and adolescents. We tested its accuracy with a public database of X-rays of children’s fractures taken during the treatment and healing process. We used part of the X-ray database for training artificial intelligence (AI, or machine learning) programs to classify stages of bone healing when using our new system. We used another portion of the same database to test the performance of the AI system that had been trained with our new classification system. Our new system addresses certain classification ambiguities of a currently used system and is similar in accuracy.

**Abstract:**

A timeline of pediatric bone healing using fracture healing characteristics that can be assessed solely using radiographs would be practical for forensic casework, where the fracture event may precede death by days, months, or years. However, the dating of fractures from radiographs is difficult, imprecise, and lacks consensus, as only a few aspects of the healing process are visible on radiographs. Multiple studies in both the clinical and forensic literature have attempted to develop a usable scale to assess pediatric bone healing on radiographs using various healing characteristics. In contrast to the orthopedic definition, a fracture in forensic casework is only considered to be healed when the area around the fracture has been remodeled to the point that the fracture is difficult to detect on a radiograph or on the surface of the bone itself, a process that can take several years. We subjectively assessed visible characteristics of healing in radiograms of fractures occurring in 942 living children and adolescents. By dividing these assessments into learning and test (validation) sets, the accuracy of a newly proposed fracture healing scale was compared to a previous study. Two machine learning models were used to test predictions of the new scale. All three models produced similar estimates with substantial imprecision. Results corroborate the Malone model with an independent dataset and support the efficacy of using less complex models to estimate fracture age in children.

## 1. Introduction

Bone fractures in children are a relatively common occurrence. Approximately one-half of girls and two-thirds of boys will have fractured a bone by the age of 15 [1]. Most childhood fractures occur either while playing sports or as a result of household accidents, while fractures as a result of inflicted injury or high-impact trauma such as vehicle accidents make up only a small portion of fractures [2,3,4]. The radius and ulna are the two bones most commonly fractured in childhood (i.e., post-infancy), accounting for approximately one-third of all fractures; fractures of the clavicle, tibia, humerus, and various bones in the hands and feet are also common [4].

The location and age of antemortem bone fractures can be used for personal identification of unidentified human remains. This is commonly achieved by comparing antemortem radiographs from the presumed individual with postmortem radiographs taken of the corresponding skeletal element. The location and type of fracture, including one that would be considered healed, can be assessed for concordance, but only a general assessment of the age of the fracture at the time of death (i.e., healed vs. healing) can be made in most cases because rates of fracture healing specific to dry bone have not been determined [5]. In addition, the currently available methods of dating fractures solely using radiographs are difficult to use, imprecise, and lack consensus about which fracture healing characteristics should be used [6,7,8,9,10]. This limits the ability to include or exclude individuals from further consideration based on antemortem fracture characteristics. When it is feasible to prepare suitable samples, histological methods can provide orthogonal information to complement that available from radiographs [5,11,12,13,14]. In cases of suspected inflicted injury, knowing how long ago a particular fracture occurred can be critical for making a criminal case against the abuser [15]. Other factors, including the type and site of fracture relative to the child’s age, also contribute to the assessment of possible abuse involving fractures [7,16,17,18].

In clinical orthopedics, there is no general agreement on the definition of a healed fracture [19]. For most patients, a fracture is considered to be healed when the bone has achieved clinical union (i.e., the patient can bear weight on the injured limb with minimal pain) and the fracture callus bridges at least three of the four bone cortices [6,9,20,21,22,23,24], a process that usually takes 8–16 weeks in adults, depending on the patient’s age and the location of the fracture [19] and 3–5 weeks in young children [25]. The time required for bone healing can be affected by the type and location of the fracture [3,26,27,28], the individual’s age and nutritional status [3,26,27,28,29], any internal or external fixation of the fracture during the healing process [30], and any underlying medical conditions [26,28,31] or concurrent injuries [32]. In children, the normal bone growth process is also thought to affect the time it takes for a fracture to heal [3,33,34].

In contrast to the clinical definition, a fracture in forensic casework is only considered to be fully healed when the area around the fracture has been remodeled to the point that the fracture is difficult to detect on a radiograph or on the surface of the bone itself, a process that can take several years [29,35]. This discrepancy between the forensic and orthopedic definitions of healing suggests that clinical models of bone healing may not be detailed or specific enough to be useful in forensic casework. In addition, as many diseases and lifestyle choices studied in the clinical literature do not visibly affect the skeleton and would not be evident on the remains of an unidentified individual [36], general models of human bone healing that take into account the age of the child and location of the fracture would be more applicable to forensic casework.

A timeline of bone healing that uses fracture healing characteristics that can be assessed using radiographs alone would be advantageous for forensic casework. Radiographs of visible fractures are commonly part of both the standard forensic anthropology and medical examiner report, providing a non-destructive method for documenting and analyzing the fracture. Radiographs can also be used to view fractures on either fleshed or skeletonized remains, which would allow for fracture age assessment in both deceased children and living children. This would be especially beneficial in cases where physical abuse of a living child is suspected. However, one obvious limitation with assessing fracture healing using radiographs is that they create a two-dimensional depiction of a three-dimensional structure. In most cases, taking multiple radiographs of each fracture from different angles can resolve this issue.

Radiographs are also limited in that they can only depict bone changes involving significant osteoblastic or osteoclastic activity, which means that only a few aspects of the healing process are visible. This makes estimating bone fracture healing using only radiographs a difficult and inexact process. The primary skeletal evidence of antemortem fracture healing visible on radiographs is the formation of a callus of new bone around the fracture site [5]. Other fracture healing characteristics, such as the shape and visibility of the fracture line and the presence/absence of sclerotic bone at the fracture margins, and sub-periosteal new bone formation (particularly in infants), may also be indicative of a timeline of fracture healing [37], but these characteristics are more difficult to observe and are less widely studied.

Immediately after a fracture occurs, and for up to 14 days afterward, the fracture line appears sharp on radiographs [27,38]. As fracture healing takes place, the fracture line becomes less well-defined on radiographs, eventually disappearing altogether as the gap between the fracture lines bridges [27,38]. This process is what is typically considered fracture union and is thought to follow a roughly log-normal distribution, with fractures in younger children taking much less time to achieve union of the fracture gap than older children [39]. A scoring system specifically for estimating fracture healing in certain long bones treated by intermedullary nailing is based on fracture line appearance and callus bridging observed at four cortices of tibia [21,22,23] or radii [24].

A brief period of widening of the gap between the fracture lines due to resorption of bone on the fracture line is thought to be another indicator of healing [27,38]. However, in most forensic casework, radiographs from only one time point would be available for analysis, and it would be impossible to accurately observe whether or not the fracture gap is wider than it was previously. Due to this, methods of fracture healing developed for forensic casework purposes should not include fracture gap widening as a healing criterion.

Increased bone density (sclerosis) on either side of the fracture line compared to that of the adjacent bone usually appears by 4–6 weeks after injury in children beyond infancy [37]. This can be seen on radiographs as a radiopaque area on or near the fracture line and, in the case of buckle or torus fractures that do not produce a visible fracture line, is sometimes the only visible sign of healing [37]. Later in the healing process, bone density around the fracture approaches the levels before the fracture occurred and the area no longer appears radiopaque compared to the surrounding bone tissue. Although the presence or absence of sclerosis could be a valuable dating characteristic for older fractures, few studies include sclerosis as a criterion for bone healing (Ref. [40] is an exception).

Multiple studies in both the clinical and forensic literature have attempted to develop a usable scale to assess pediatric bone healing on radiographs using various healing characteristics [25,29,37,38], but it is generally acknowledged that such categorizations are imperfect: “the healing process is … separated only arbitrarily into phases for the purposes of study … and such separation is an abstraction” [41], p. 203 and “these stages are not discrete and exist on more of a continuum… ” [16], p. 149. These studies all suffer from small sample sizes and limited participant age ranges and/or fracture locations; consequently, the fracture age ranges developed from these studies (when reported) are very large, to the point of being nearly useless in a forensic investigation. Although largely anecdotal [10], experts agree that younger children heal faster than older children and adults [3,29,33,34,42,43]. Studies usually group children of different ages into arbitrary age groups, and only one attempt has been made to include children older than age six in studies of fracture healing [37]. With the exception of the study by Malone et al. [29], all of the studies also incorporate at least one fracture healing stage or criterion that relies on having previous radiographs of the fracture available for examination, making them inapplicable in much of forensic case work.

Currently, the study of bone fracture healing in children by Malone et al. [29] is most applicable to forensic casework. They used clinical radiographs of radius and tibia fractures with a known fracture date from 107 children ages 0–5 taken at multiple time points to develop a stage system for assessing the age of fractures and time since injury. Malone et al. assessed fracture healing using six stages of fracture healing (Table 1). The results from the Malone et al. study indicate that the earlier stages of fracture healing roughly corresponded to a 2–8-week time period in the fracture healing process, but the time period for each stage overlapped significantly with the time periods for other stages. The fracture age ranges observed for each stage were also large, especially for stages 5 and 6.

Although the Malone et al. [29] study suggests that it may be possible to predict how long ago a fracture occurred using healing stages, the criteria for several of the healing stages lack objectivity (e.g., callus presence minimal, fracture line significantly blurred), or are equivocal, with some stages appearing to contradict information presented in earlier stages (i.e., stage 2: blurring of fracture line vs. stage 3: fracture line visible but may be blurred). The small sample size, restricted age ranges, and a lack of standardization based on fracture location limit the applicability of the results. Larger sample sizes that contain a broader age range of participants and a wider variety of fracture locations are needed to develop fracture healing timelines that are both accurate and precise.

Given the limitations of current fracture healing studies, the intent of the present study is twofold: to assess the accuracy and reliability of using stages or characteristics of bone healing visible on clinical radiographs of living children to predict when the fracture may have occurred and to develop an alternative timeline of fracture healing for children based on radiographs that could be applied to both antemortem and postmortem examinations.

See the end of the document for further details on references.

## 2. Materials and Methods

**Figure 1:**
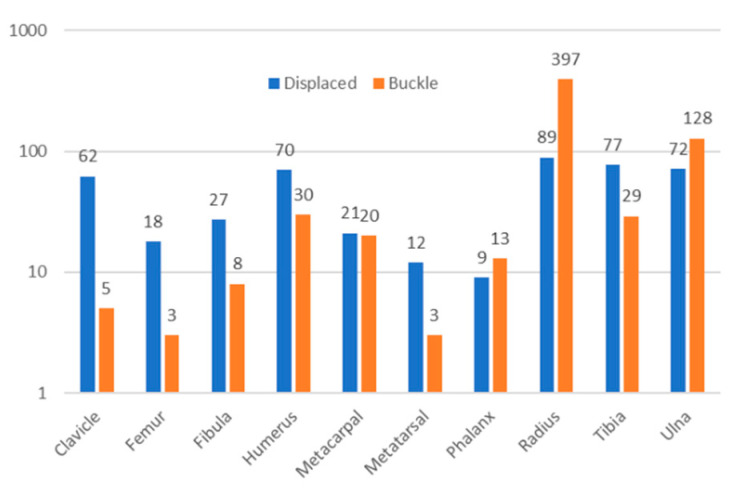
Number of fracture incidents by location (log scale) in displaced (blue) and buckle (orange) fractures.

**Figure 2:**
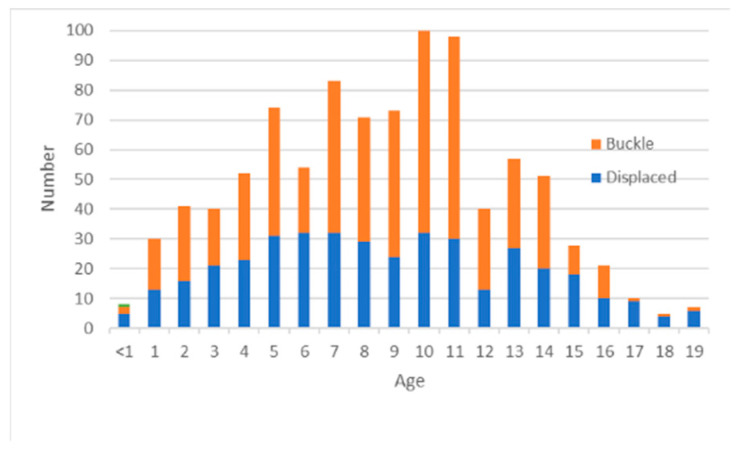
Distribution of patient age and fracture type in displaced (blue) and buckle (orange) fractures.

Radiographs were obtained from the PATRICIA children’s radiograph collection [44]. This publicly available, anonymized online database consists of both medicolegal and clinical radiographs, originally intended for use in age estimation studies. The FBI Laboratory Institutional Review Board approved use of this dataset and supplementary injury date information (docket 420-17; approved 14 December 2017). Only clinical radiographs were utilized for this study because time-since-fracture information is often unavailable for medicolegal radiographs. Information about occurrence of child abuse was unavailable. Poor quality radiographs, radiographs without a visible fracture, and records removed from consideration due to statistical and clinical irregularities (see below) were likewise removed from further consideration, resulting in a dataset of 1813 fracture time point records (927 displaced, 886 buckle) from 942 fractures (395 displaced, 547 buckle) occurring in various bones (Figure 1) in individuals ranging in age from 0–19 year (Figure 2). The predominance of buckle fractures of the distal radius shown in Figure 1 is consistent with the typical mode and location of childhood fractures [1,3,4,20,42,45]. The distribution of patient ages in this convenience dataset is centered in mid-childhood (Figure 2).

The sex and exact age of each individual with a visible fracture was recorded, along with the location of the fracture. Any visible surgical internal fixation devices (e.g., pins, rods, or screws) were also noted. Date of each injury (i.e., first radiograph), not available in the online version of the PATRICIA radiograph collection, was obtained from the orthopedic clinic that originally provided the clinical radiographs for the PATRICIA collection. Fracture age was calculated for each healing time point as the time elapsed since the first radiograph, as exact date of trauma was unavailable.

There is widespread agreement that characteristics of bone healing are a continuum with considerable overlap rather than discrete stages [10]. Based on the results of previously published fracture healing studies, we developed scoring descriptions to independently classify the appearance of the fracture callus, fracture line/gap, and sclerosis for each fracture, as an alternative to combining all three criteria into a limited number of discrete stages. As most buckle fractures are characterized on radiographs by a bone discontinuity rather than a distinct fracture line or gap, a separate fracture discontinuity criterion was defined to use for buckle fractures in place of the fracture gap criteria. The proposed criteria descriptions are listed in Table 2. To compare the efficacy of the proposed fracture healing criteria with the currently available forensic method, every fracture time point in the database was scored by both methods. Illustrative radiographs of a radius and/or ulna (Supplementary Figures S1–S18) depict each of the fracture healing criteria defined in Table 2, as well as the Malone et al. stage of healing (Table 1) to which it was assigned. A single forensic anthropologist (K.K.) performed all examinations. Limited intraobserver repeatability was tested by conducting a second assessment, separated by time, in some cases. Assessment of a very large number of radiographs is an arduous and time-consuming task. Several authors have reported high levels of inter- and intra-observer reproducibility among expert assessors of fracture healing. Stagings illustrated by Figures S1–S18 were reviewed by a second forensic anthropologist.

To assess the accuracy and precision of the fracture healing criteria described in Table 1 and Table 2, the displaced and buckle fracture datasets were each randomly divided into a training dataset consisting of 70% of the data and a test (validation) dataset comprising 30% of the data. The datasets contained information on healing time, subject age, subject gender, fracture location, and subjective assignments to the criteria in Table 1 and Table 2. For the newly proposed criteria in Table 2, the training dataset was used to develop two supervised machine learning models to predict fracture healing in children, while the test dataset was used to validate the accuracy and precision of the predictive models. The Malone et al. [29] model was evaluated by comparing assessments within the same training and test datasets. Multivariate linear regression was employed to explore the correlation between fracture age, patient age, and Malone stage. Initial attempts to develop a multivariate least squares regression model involving all the dataset variables were abandoned due to its poor performance.

Prior to fitting the machine learning models, the healing time data were log-transformed to improve their homoscedasticity. Two machine learning models, characterized by their flexibility and exceptional predictive ability when applied to multivariate data, were chosen. Moreover, the models used are essentially more sophisticated variants of the basic decision tree employed by Malone et al. [29].

The first approach employed a random forest model. The method works by repeatedly, randomly selecting subsets of the available predictor variables then using bootstrapped samples from the training dataset to construct a series of decision trees. The individual trees are than combined to produce an overall model that provides more accurate predictions than any single decision tree could. Model fits are aimed at minimizing the Mean Square Error (MSE) or equivalently the Root Mean Squared Error (RMSE) as measured using the Out of Bag sample. Hastie et al. provide a detailed discussion of random forest models for the interested reader [46].

It is necessary to pre-tune the model for optimal performance. The random forest model was fitted using R version 3.5.2 software and the Ranger package [47,48]. Important adjustable parameters in the Ranger package include the fraction of the test sample used to fit the model (i.e., the within bag sample), the number of predictor parameters to be randomly sampled from the total number available, the minimum number of samples points per node and the total number of trees to be used in fitting. Tuning parameters for the random forest model were optimized using a hypergrid of candidate parameter values to select an optimized set.

Once an optimal (or near optimal) set of parameters is identified, a final model using the selected parameters is created and fit using the training dataset. Monitoring of the reduction in mean squared error as each predictor variable is repeatedly used as a node for branching across the individual decision trees provides a way to estimate its relative contribution to the overall reduction in variance. A basic R program used for tuning and implementing the random forest model is provided in Supplementary File S1. Additional details on implementation of the Ranger program are available [49]. A gradient boosting machine (GBM) model was also fitted to the training dataset. In contrast to random forest models, GBMs are slow learning models [50,51]. GBMs build an ensemble of shallow and weak successive trees. Each individual tree explains only a small portion of the total data variance. Every subsequent tree learns from and improves upon the previous one by modeling a small portion of the remaining, unexplained variance. When combined, these many weak, successive trees can produce a single powerful prediction model. The GBM model was fit with the GBM package in R [52]. Important tuning parameters for the model include: the number of trees, the depth of the trees (number of splits in each tree), the learning rate (gradient descent rate), and the fraction of training data (subsampling of training data) used for fitting. Tuning parameters for the GBM model were optimized using a hypergrid of candidate parameter values to select an optimized set. The basic R program used for tuning and implementing the GBM model is provided in Supplementary File S2. A greatly expanded discussion of the process used to implement GBM models is available [53].

Patient age at the first visit to the clinic was used to establish the starting point of treatment, which, it is crucial to note, might be days or weeks later than the actual trauma. Radiographs recorded during the initial clinic visit were not used for modeling or staging. Potential outliers were identified from the results of Malone et al. [29] staging as (Q3 + 3 × IQR) and (Q1 − 3 × IQR), where IQR is the interquartile range, Q1 is the first data quartile, and Q3 is the third quartile [54]. All of the outliers that met these criteria occurred within the upper outer fence, i.e., (Q3 + 3 × IQR). Decisions about which of these potential outliers could justifiably be removed are discussed and tested in the Results section below.

## 3. Results

**Figure 3:**
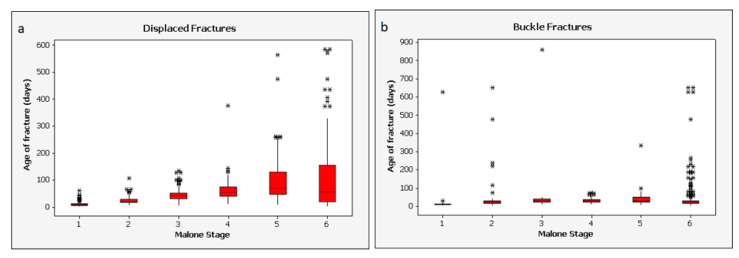
Descriptive plots of the fracture datasets, by Malone stage: (**a**) displaced, (**b**) buckle fractures.

Descriptive plots of the entire dataset after assignment to the Malone et al., scale [29] reveal data variability and the presence of many presumptive outliers (Figure 3). Both factors must limit the potential precision achievable with any predictive model.

Decisions to remove statistical outliers, particularly from clinical datasets, are fraught. If attempted at all, each removal obliges justification. In practice, there is no way to predict that the case at hand may be an outlier, which mitigates against removing them from the training set. On the other hand, we took the position that it is useful to model the more typical situations. Of contingent outliers statistically identified within the two datasets, a few were judiciously removed due to absence of healing after a prolonged period: of 18 candidates, 5 were removed from the displaced dataset, and of 37 suspect buckle fractures, 1 was removed. Among records identified as suspicious due to being assigned to Malone stage 5 or 6, despite being 14 days old or younger, additional records were culled: of 38 candidates among displaced fracture records, 2 were removed, and of 91 buckle fractures, 2 were removed. As a result, the datasets were reduced to 927 displaced and 886 buckle fracture records.

**Figure 4:**
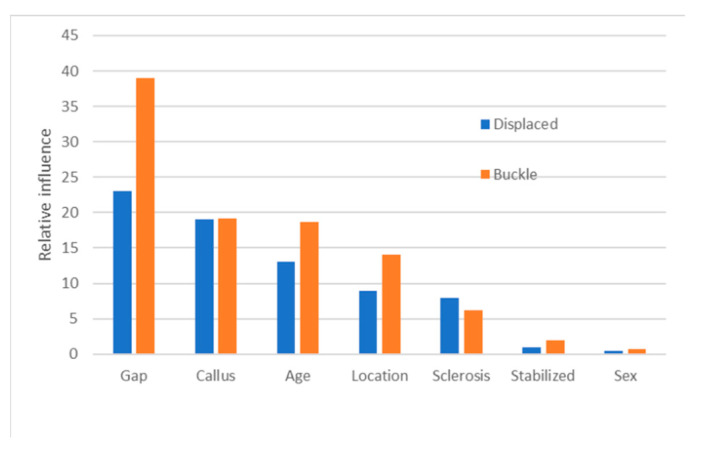
Relative contribution of variables to variance in displaced (blue) and buckle (orange) fractures.

For both the Malone fracture age scale (Table 1) and the newly proposed scale (Table 2), the same test sets were used to compare the value predicted by each method to the observed value of each data point. The absolute difference between the predicted and observed values was used as a measure of each model’s precision. For example, if the predicted value is 10 days and the observed value is 8 days, the absolute difference is 2 days. Absolute differences are used in preference to simple difference because each model produced a differently biased mean value for the simple differences, which made it more difficult to compare models. The reduction in MSE for each variable was accumulated for each variable in the random forest model. For both displaced and buckle fractures, fracture gap, callus, and patient age tended to be most predictive of fracture age; sex and fixation were least so (Figure 4). In contrast to the Malone et al. study [29], fracture location was of lesser predictive importance.

**Figure 5:**
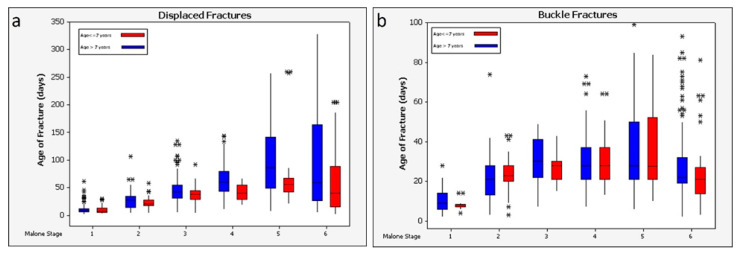
Descriptive plots of the fracture datasets, by Malone stage, and grouped by patients age 7 and younger (red) and those older than 7 (blue). (Note: recorded fracture ages greater than 350 days are truncated for clarity). (**a**) displaced, (**b**) buckle fractures.

Dependence of healing time on subject age was further explored via multivariate linear regression of fracture age (healing time point) vs. two independent variables, patient age and Malone stage. For both buckle and displaced fractures, correlation with the Malone stage was significant (*p* < 0.001). Age of patient was significantly correlated for displaced fractures (*p* < 0.001) but not for buckle fractures (*p* = 0.15) (note that the assumption of homoscedasticity is tenuous, particularly for buckle fractures). The potential correlation of patient age with healing time was further assessed via box plots depicting times recorded at each of the six Malone stages, partitioned by patients older than 7 and those aged 7 and younger (Figure 5).

Table 3 presents the median and mean fracture age within the two test datasets as predicted by the Malone et al. approach [29]. The estimated age of a fracture is simply the median or mean of the data from the test set corresponding to a particular level of the scale. The presence of many outliers in the data whose removal cannot reasonably be justified indicates that medians better represent central tendency than do the means, particularly for the higher stages of healing (Figure 5).

The median and mean differences between the predicted fracture age and the true fracture age using the Malone et al. [29] criteria, and the proposed criteria classified using a random forest model and a GBM model, are compared in Table 4. The same datasets were used for training each model and testing the resulting predictions. On a global basis, median differences between predicted and true value would indicate that the proposed scale and classifiers produced more accurate estimates of fracture age than the Malone scale (median being more informative than means for these highly skewed data). The high variability in individual fracture age estimates clarifies that there is little practical difference between the two scales. For displaced fractures, the GBM model (mean difference between predicted and true value, −9 days) and the random forest model (mean difference, −10 days) slightly underestimated the true age of the fracture on average, while the Malone model (mean difference, 0 days) did not, on average. For buckle fractures, the Malone model underestimated the true age of the fracture by 6 days, while the GBM and random forest models underestimated it by 14 days when results are averaged across all stages (Malone) or conditions (GBM and Random Forest). For both displaced and buckle fractures, mean differences from the GBM and the random forest predictions are not significantly different from those predicted by the Malone model (*p* > 0.05). Similar calculations conducted before outlier removal produce results that are not significantly different from those in Table 3 and Table 4 (*p* > 0.5, data not shown), indicating that removal of the extreme outliers had a negligible effect on model outcome. The standard deviations of the mean differences for all tested models are very large, ranging from 52 days for displaced fractures using the GBM model to 82 days for buckle fractures using the Malone model. These high uncertainties reflect the individual variations in healing and any limitations in the available data.

**Figure 6:**
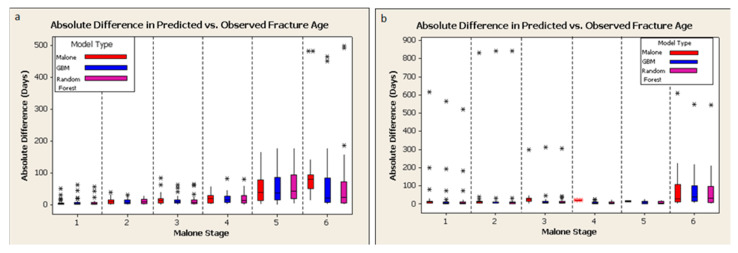
Absolute error in fracture age as predicted by the Malone (red), GBM (blue), and random forest (violet) models, depicted via the six Malone stages: (**a**) displaced, (**b**) buckle fractures.

Descriptive plots illustrate more fully the comparable performance of the three models (Figure 6). Reflective of the variations in both datasets (Figure 3 and Figure 5), outliers are apparent. The plots of absolute differences do not support a strong difference in predictive value among the three model types. The two alternative models do not obviously outperform the Malone scale. This may be due to the outliers in the dataset, or it may imply that additional variables carry no more predictive information than the simpler Malone scale.

## 4. Discussion

Radiographic methods of fracture healing are convenient to perform, but they are limited in that only a few signs of fracture healing are observable on radiographs. Combining a radiographic method with other methods of fracture healing assessment, such as computed tomography [55] or direct observation of fracture healing on de-fleshed bone, may be a way to increase accuracy and precision of fracture healing assessment in forensic casework. These methods would potentially be more time-consuming and costly than radiographic methods but would allow for better visualization of the surface-level healing process [56].

Displaced, but not buckle, bone fractures in children under the age of 7 healed significantly faster than fractures in children older than 7 (*p* < 0.001). This is consistent with findings from previous studies indicating that younger children heal more quickly than older children [3,29,33,42,57], but a direct comparison of results is not possible because only one study includes children over the age 6 in its samples [8]. Descriptive plots comparing Malone staging of fractures in children younger and older than age 7 (Figure 5) showed moderate differences, but not enough to justify separate models for these two cohorts.

Although the age of a child can be accurately assessed from skeletal remains, sex and ancestry cannot accurately be assessed in children using skeletal morphology until the late teenage years [58,59,60]. During data analysis, we included sex as a factor with the intent of potentially developing both sex-specific and combined sex models, but as differences in healing times between males and females contributed only marginally to variance (Figure 4), the combined-sex models were deemed sufficient.

Ancestry could not be included as a factor in the models in this study because the ancestry of most of the individuals in the dataset was not known. Two studies reported no effect on healing of ethnicity or socio-economic group [42,61]. Neglecting ancestry as a factor is also reasonable given the difficulty in assessing ancestry from the skeletal remains of children.

Although most clinically treated fractures will be stabilized using either external fixation (a cast and/or sling) or internal fixation (a surgical rod or pin) at some point in the fracture healing process, it is typically not possible to verify whether a fracture in a forensic case was externally stabilized in the past unless the cast or sling is in place at the time of exam. In contrast, internal fixation devices are visible on radiographs and will often be left in place long after the healing process is declared complete, making them potentially useful for both fracture healing time modification and positive identification efforts. The presence or absence of internal fixation was included as a factor in data analysis, but no significant differences in healing times between internally fixed and unfixed fractures were observed. The results from random forest modeling support that fixation was a minor factor (Figure 4). This finding contradicts previous studies [30,62] suggesting that internal fixation affects healing rate, but the inconsistency may be due to differences in how fracture healing is scored between studies. Internal fixation may restrict radiographic characterization of the healing process if the fracture gap and callus are not visible [62].

An issue with using the Malone stages (Table 1) to assess fracture healing is that the stages rely on observations of both a fracture callus and a visible fracture line, but the latter is rarely observed with buckle fractures. While scoring the fractures using both scoring systems, buckle fractures tended to be scored as a Malone stage 5 or 6 throughout the healing process, making the Malone stages essentially useless for buckle fractures. Figure 5 reveals another anomaly with the buckle fracture data. The median healing time for Malone stage 6 patients is lower than that for stage 5. Undoubtedly, data quality is an issue, as is natural human variation, and consequent outliers continue to confound the analysis. Malone et al. also reported substantial imprecision in fracture age estimates for stages 5 and 6 [29]. As observed by Malone et al., stage 6 is problematic because complete healing has no defined end point.

Buckle fractures are common in children and comprise approximately 50% of the fractures observed in our study dataset, so it is necessary to include a method of scoring buckle fractures in any staging or criteria-based system of fracture healing. Scoring each fracture healing criterion separately and adding a “fracture discontinuity” criterion for buckle fractures to be used in place of the “fracture gap” healing criterion reduces this problem somewhat, but creates a separate problem in that it can sometimes be difficult to differentiate between the two fracture types, especially during the later stages of healing. Clinical studies acknowledge that fracture dating becomes less precise the longer after trauma it is observed [17,33]. During the scoring phase of the project, some of the oldest displaced fractures in the dataset were originally misclassified as buckle fractures at later fracture time points and had to be rescored after the fact. In forensic casework, rescoring the fracture would not be feasible in many cases due to a lack of prior information about the fracture, leaving anthropologists and medical examiners to use their best judgement in assessing the fracture. However, because the criteria and stages associated with the latter parts of the healing process also have very wide confidence intervals, misclassifying a late-stage displaced fracture as a buckle fracture (or vice versa) is unlikely to cause significant differences in estimating the age of a fracture.

Another difficulty with separate scoring criteria instead of a combined stage system is that two of the criteria (callus appearance and sclerosis) will be scored as level 1 both immediately after the fracture and after the fracture has healed completely. This is unavoidable, because visible fracture callus and visible sclerosis are absent in completely healed as well as in completely unhealed fractures. In most situations, it is relatively easy for an observer to determine whether a fracture is completely healed vs. completely unhealed by looking at the appearance of the fracture line/gap, but few extant statistical methods account for this situation well. This also means that it is difficult to truly assess each healing criterion independently, as the fracture gap healing criterion is ultimately being used to determine whether a lack of fracture callus and/or sclerosis is indicative of an early stage fracture or a late-stage fracture, even though the criteria are being scored independently of each other.

Numerous complications are inevitably associated with a study using uncontrolled clinical data. Recorded time points all represent one point in a continuum, i.e., when the patient is seen, and where they happen to be relative to a Malone stage or combination of healing characteristics. The date of the first X-ray (Day 0) could be some time after the trauma, disrupting the timeline on which we based our models. Relative to displaced fractures, initial treatment of buckle fractures may be delayed because the injury may seem less serious at first. Even if Day 0 occurs soon after the trauma, the patient may not be seen again for weeks, casting uncertainty on what population is represented by stage 1. Depending on the severity and complexity of the fracture, radiographs may not be taken during the middle stages of the fracture healing process, missing some stages entirely. Additionally, fractures at Malone stage 6 are unlikely to be X-rayed as soon as they are completely healed and would be assigned that stage for an unlimited time.

Although every effort was made to verify anomalous values over the course of this study, there is always the possibility of some degree of data error in any retrospective study. As this particular study involved patient records, the possibility exists that a clinician may have inadvertently recorded the date of fracture incorrectly during the intake exam for some of the records. It is also possible that a patient or patient’s guardian may have misreported the date of fracture to the clinician in some cases, either purposely (e.g., for insurance coverage reasons or to hide physical abuse) or accidentally (misremembering the date of injury). Only limited estimation of intra- and inter-observer reproducibility of fracture healing was conducted, although other studies reported good agreement [4,18,34]. If these errors affect some of the records in this study, they would likely have the effect of decreasing precision of the model by widening the confidence interval range for a prediction rather than artificially increasing precision by narrowing the range of the confidence interval.

## 5. Conclusions

Scoring bone fractures using separate healing criteria can give a general fracture age range, but the accuracy and precision of using separate healing criteria is similar to that of stage-based methods for the fracture dataset used in this study. The predictive accuracy of all the methods was poor. The plots of the absolute differences from observed ages of displaced and buckle fracture data do not seem to support a strong difference in predictive value among the three model types (Figure 6). Given that they are simpler to score and analyze, we recommend using a stage-based system of fracture healing such as the Malone et al. method [29] when estimating ages of children’s fractures. Within this cohort, patient age and sex were not highly predictive of fracture age. Future research should explore combining separate fracture healing criteria on radiographs with direct observation of healing on the surface of a bone to increase accuracy and precision of fracture age estimation. Revisiting the characteristics defining the Malone stages could perhaps mitigate some of the ambiguities we identified.

## Figures and Tables

**Table 1:** Malone et al. [29] stages and their descriptions.

Stage	Stage Description	Mean Healing Time (Days)	Range	SD
1	No healing: sharp fracture lines, absence of bridging and callus formation	3.3	0–14	3.4
2	Granulation: beginning of resorption along fracture line, “fluffy” callus formation, blurring of fracture line, absence of a complete mature callus	21	4–50	10.5
3	Callus: mature callus formation around fracture site; callus bulging over site and demonstrating a radiopaque appearance, fracture line visible but may be blurred	38.4	15–75	13.4
4	Bridging: fracture gap is connected across the fracture site in some, but not all areas (<50%), blurring of the fracture line, callus may still be present	43.9	24–93	15.2
5	Clinical Union: fracture line is significantly blurred; fracture line is connected in most areas (more than 50%), callus presence minimal	65.2	24–156	48.2
6	Completion: no evidence of fracture line, callus presence minimal or not observable	313.3	42–750	235.7

**Table 2:** Newly developed criteria used to score fracture healing in this study.

Criterion	Score	Description
	1	No visible fracture callus
Callus appearance	2	Fracture callus is visible, but is not the same radiodensity throughout and appears wispy, patchy, or hollow in areas
	3	Fracture callus is the same radiodensity throughout, but is radiolucent compared to the unaffected bone cortex
	4	Fracture callus and unaffected bone cortex are the same radiodensity, callus is still clearly visible
Fracture discontinuity	1	Fracture discontinuity is clearly visible
(nondisplaced torus/buckle fractures)	2	Fracture discontinuity is not visible
Fracture gap bridging	1	No bridging of the fracture gap
(displaced fractures)	2	Fracture gap is bridging or bridged, but still visible
	3	Fracture gap is not visible
	1	No visible sclerosis
Sclerosis	2	Sclerosis is visible above and/or below the fracture site as a thin, roughly linear band
	3	Small patchy areas of sclerosis visible above and/or below the fracture site
	4	Widespread sclerosis above and/or below the fracture site

**Table 3:** Observed and predicted ^1^ median and mean age of fractures, as estimated by the Malone et al. [23] approach, days.

Malone Stage	1	2	3	4	5	6
**Displaced fractures**						
median observed in training set (predicted, n = 651)	8	25	41	54	65	58
median observed in test set (n = 278)	8	21	41	57	92	51
bias of prediction via median ^2^	0	−4	0	3	27	−7
mean observed in training set (predicted, n = 651) ^3^	10 (8)	26 (14)	45 (22)	63 (48)	94 (97)	103 (121)
mean observed in test set (n = 278) ^3^	11 (10)	22 (12) ^4^	42 (20)	58 (24)	118 (76) ^4^	99 (138)
bias of prediction via mean ^2^	1	−4	−3	−5	24	−4
**Buckle fractures**						
median observed in training set (predicted, n = 623)	8	22	28	28	28	22
median observed in test set (n = 265)	8	21	34	27	34	21
bias of prediction via median ^2^	0	−1	6	−1	6	−1
mean observed in training set (predicted, n = 623) ^3^	10 (5)	36 (82)	29 (10)	32 (14)	35 (20)	40 (80)
mean observed in test set (n = 265) ^3^	39 (135) ^4^	30 (41)	77 (190) ^4^	28 (11) ^4^	47 (52) ^4^	39 (72)
bias of prediction via mean ^2^	29	−6	48	−4	12	−1

^1^ Predictions are modeled from the training set (70% of dataset). ^2^ Bias of prediction is the difference: (values observed in test data) − (values predicted by training data). ^3^ Standard deviation of the mean in parentheses. ^4^ Significantly different from predicted mean, *p* < 0.001.

**Table 4:** Performance of three models: (predicted fracture age) − (true fracture age), days.

Fracture Type	Malone Scale	Proposed Scale	
		GBM	Random Forest
		Model *	Model *
Displaced (test set, n = 278)			
median difference	4.1	0.7	0.6
mean difference	0.3	−8.7	−9.6
standard deviation	56.3	52.3	55.0
Buckle (test set, n = 265)			
median difference	10.9	−0.4	−0.1
mean difference	−5.6	−14.3	−13.6
standard deviation	82.1	78.2	76.5

* Datasets were split into 70% training data and 30% test data. The same sets were used for training each model and testing the resulting models.

## Data Availability

The publicly archived dataset used in this research is available at: https://www.statsmachine.net/databases/radiographic_database/ (accessed on 18 April 2022).

## Supplementary Materials

The following supporting information can be downloaded at: https://www.mdpi.com/article/10.3390/biology11050749/s1, Figure S1: Callus stage 1: 11-day-old displaced radius and ulna fractures (scored as Malone stage 1); Figure S2: Callus stage 1: 473-day-old displaced radius fracture (scored as Malone stage 6); Figure S3: Callus stage 2: 22-day-old displaced radius and ulna fractures (scored as Malone stage 2); Figure S4: Callus stage 3: 68-day-old displaced radius and ulna fractures (ulna: scored as Malone stage 4; radius: scored as Malone stage 5); Figure S5: Callus stage 4: 176-day-old displaced radius fracture (scored as Malone stage 5); Figure S6: Callus stage 4: 89-day-old displaced radius and ulna fractures (ulna: scored as Malone stage 6; radius: scored as Malone stage 5); Figure S7: Fracture discontinuity stage 1: 0-day-old radius and ulna buckle fractures (scored as Malone stage 6); Figure S8: Fracture discontinuity stage 1: 24-day-old radius buckle fracture (scored as Malone stage 2); Figure S9: Fracture discontinuity stage 2: 108-day-old radius buckle fracture (scored as Malone stage 6); Figure S10: Fracture gap bridging stage 1: 11-day-old displaced radius fracture (scored as Malone stage 1); Figure S11: Fracture gap bridging stage 1: 0-day-old displaced radius and ulna fractures (scored as Malone stage 1); Figure S12: Fracture gap bridging stage 2: 31-day-old displaced radius and ulna fractures (scored as Malone stage 3); Figure S13: Fracture gap bridging stage 2: 59-day-old displaced radius and ulna fractures (ulna: scored as Malone stage 4; radius: scored as Malone stage 6); Figure S14: Fracture gap bridging stage 3: 204-day-old displaced radius and ulna fractures (scored as Malone stage 5); Figure S15: Sclerosis stage 1: 11-day-old displaced radius and ulna fractures (scored as Malone stage 1); Figure S16: Sclerosis stage 2: 29-day-old displaced radius fracture (scored as Malone stage 2)Figure S17: Sclerosis stage 2: 37-day-old displaced radius fracture (scored as Malone stage 2); and Figure S18: Sclerosis stage 3: 95-day-old displaced radius fracture (scored as Malone stage 6); N.B. all are in the public domain (Ousley SD. Patricia (Pediatric Radiology Interactive Atlas) https://www.statsmachine.net/databases/radiographic_database/; 2014); File S1: An R script to implement a random forest model using the Ranger package; File S2: An R script to implement a Gradient Boosting Machine model using the gbm package.
