# Supplementary material for: Postmortem and Antemortem Forensic Assessment of Pediatric Fracture Healing from Radiographs and Machine Learning Classification
Source: Biology (Basel). 2022 May 13;11(5):749. doi: 10.3390/biology11050749 (PMC9138832; doi:10.3390/biology11050749)

biology

MDPI

Supplementary figures

N.B. all are in the public domain (Ousley SD. Patricia (Pediatric Radiology Interactive Atlas) https://www.statsmachine.net/databases/radiographic_database/ ; 2014.)

Figure S1 *Callus stage 1: 11-day-old displaced radius and ulna fractures (scored as Malone stage 1)*

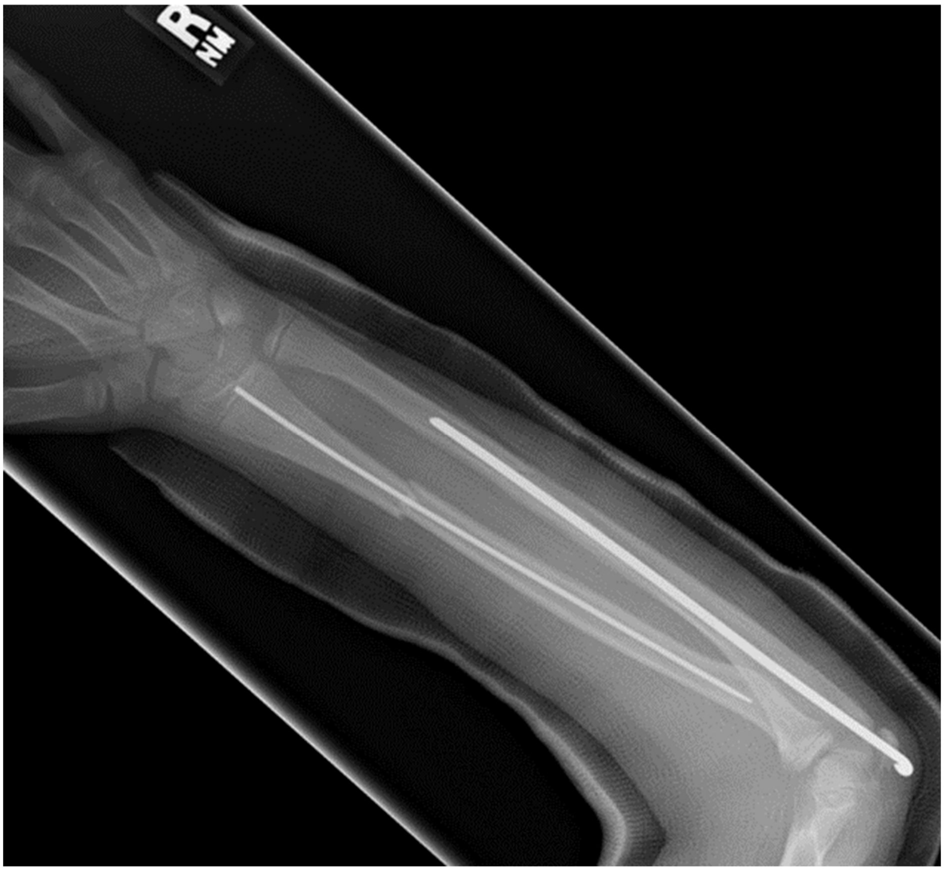

Figure S2 *Callus stage 1: 473-day-old displaced radius fracture (scored as Malone stage 6)*

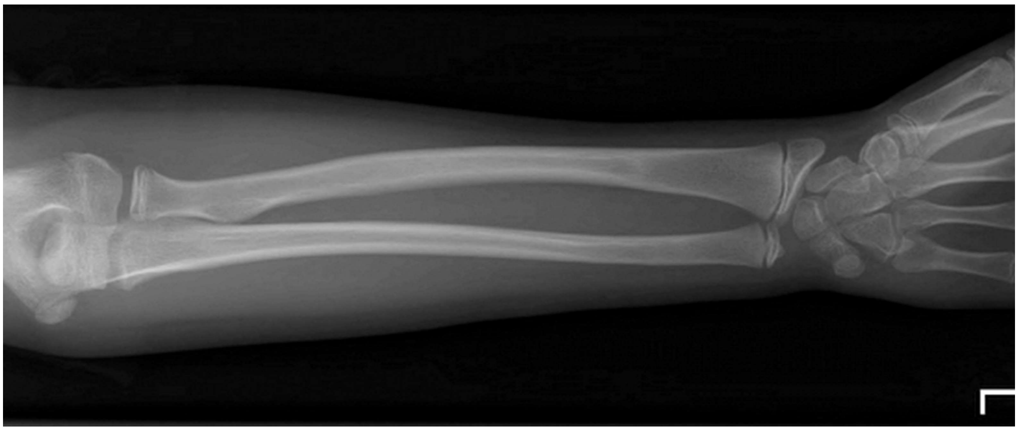

Figure S3 *Callus stage 2: 22-day-old displaced radius and ulna fractures (scored as Malone stage 2)*

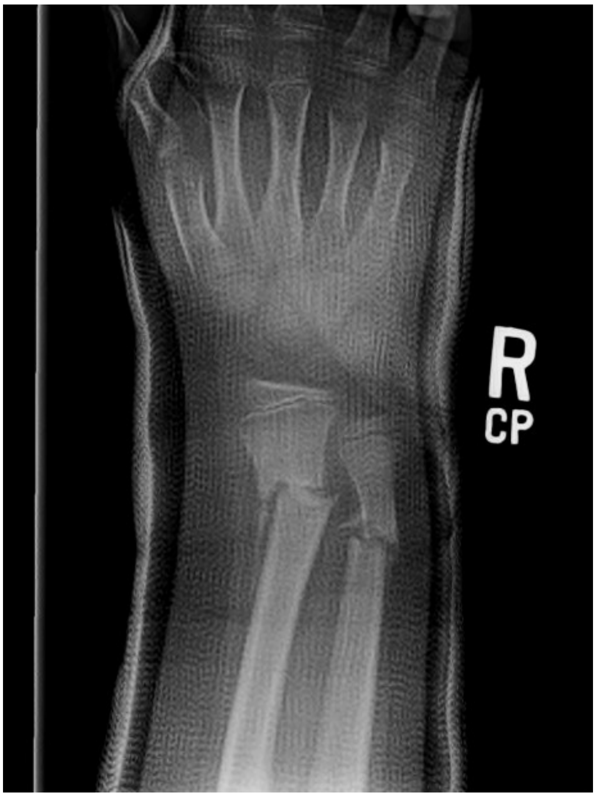

Figure S4 *Callus stage 3: 68-day-old displaced radius and ulna fractures (ulna: scored as Malone stage 4; radius: scored as Malone stage 5)*

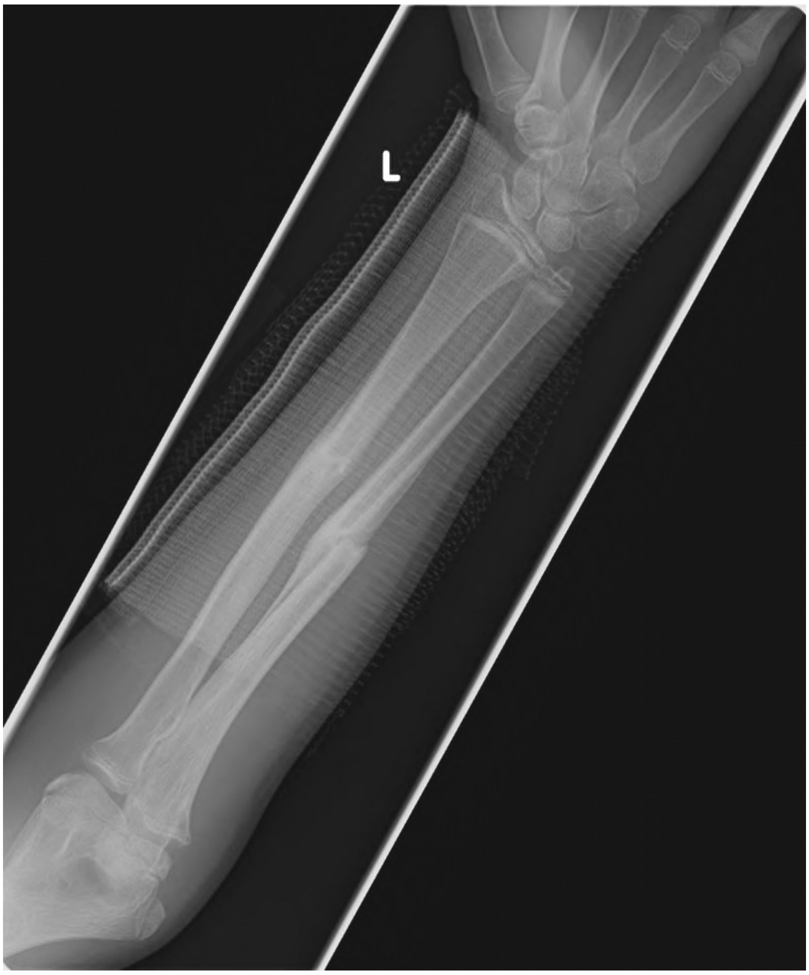

Figure S5 *Callus stage 4: 176-day-old displaced radius fracture (scored as Malone stage 5)*

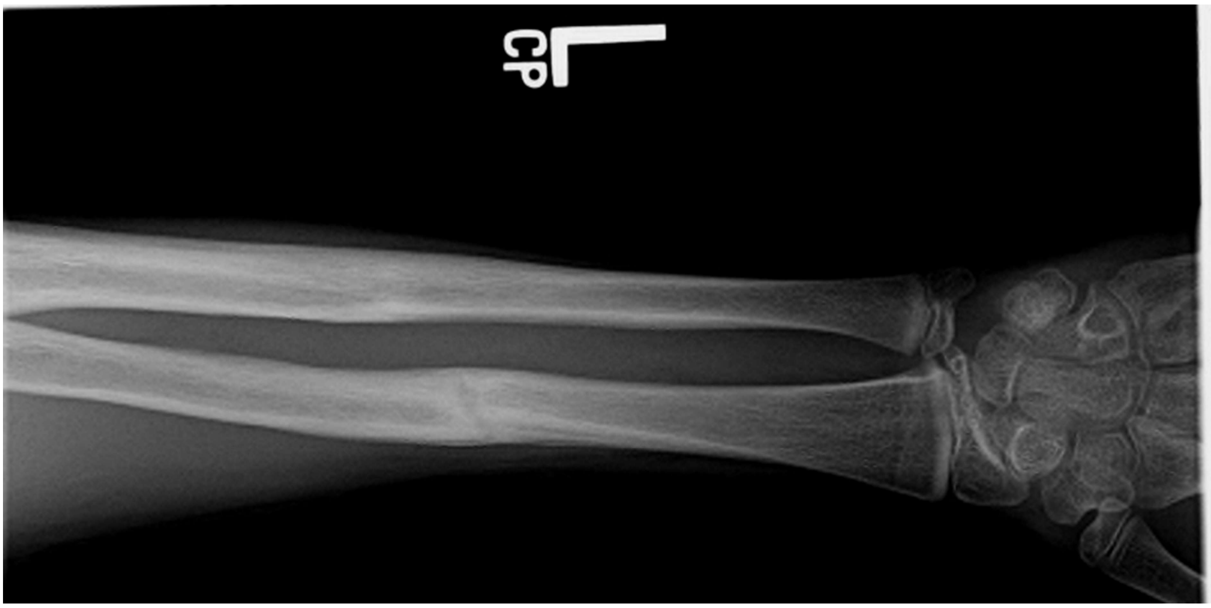

Figure S6 *Callus stage 4: 89-day-old displaced radius and ulna fractures (ulna: scored as Malone stage 6; radius: scored as Malone stage 5)*

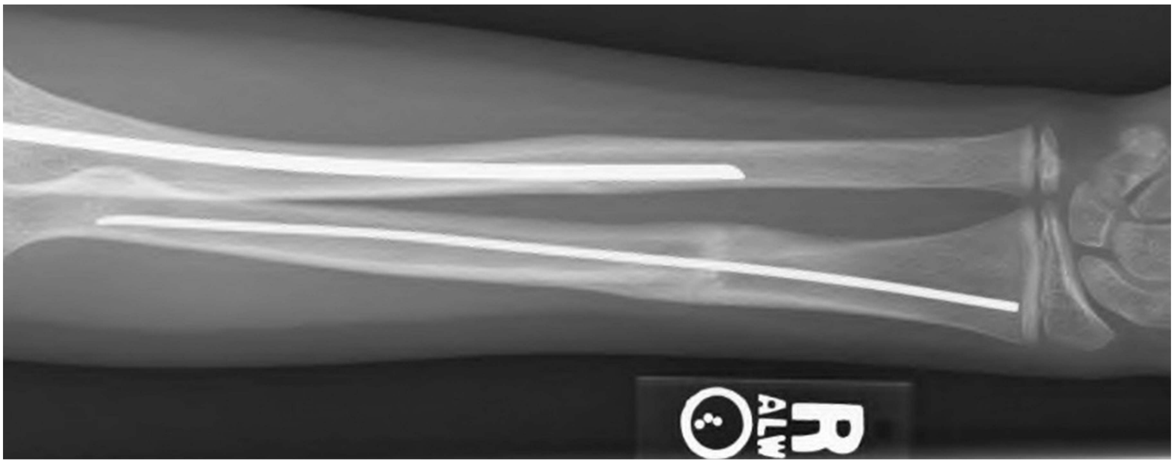

Figure S7 *Fracture discontinuity stage 1: 0-day-old radius and ulna buckle fractures (scored as Malone stage 6)*

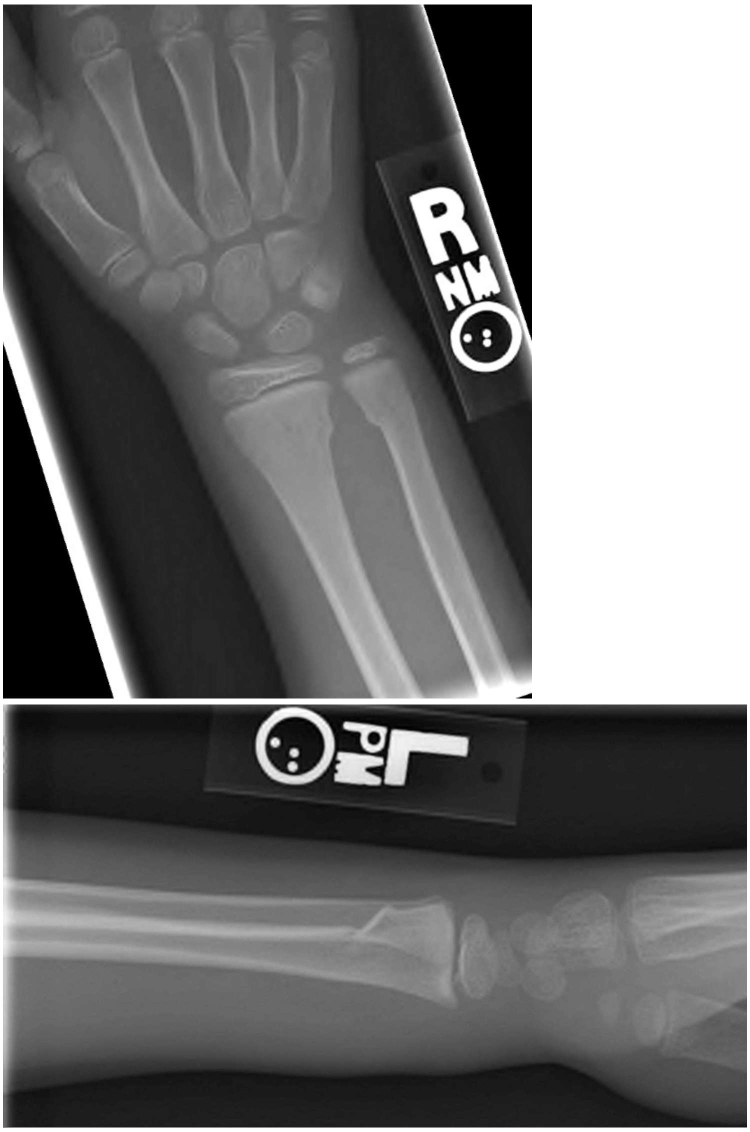

Figure S8 *Fracture discontinuity stage 1: 24-day-old radius buckle fracture (scored as Malone stage 2)*

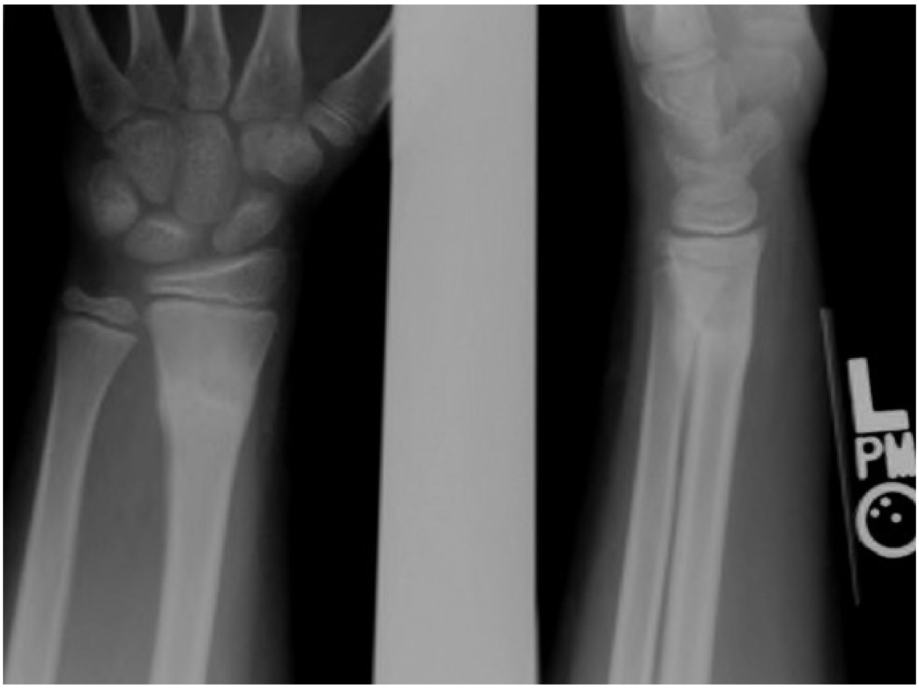

Figure S9 *Fracture discontinuity stage 2: 108-day-old radius buckle fracture (scored as Malone stage 6)*

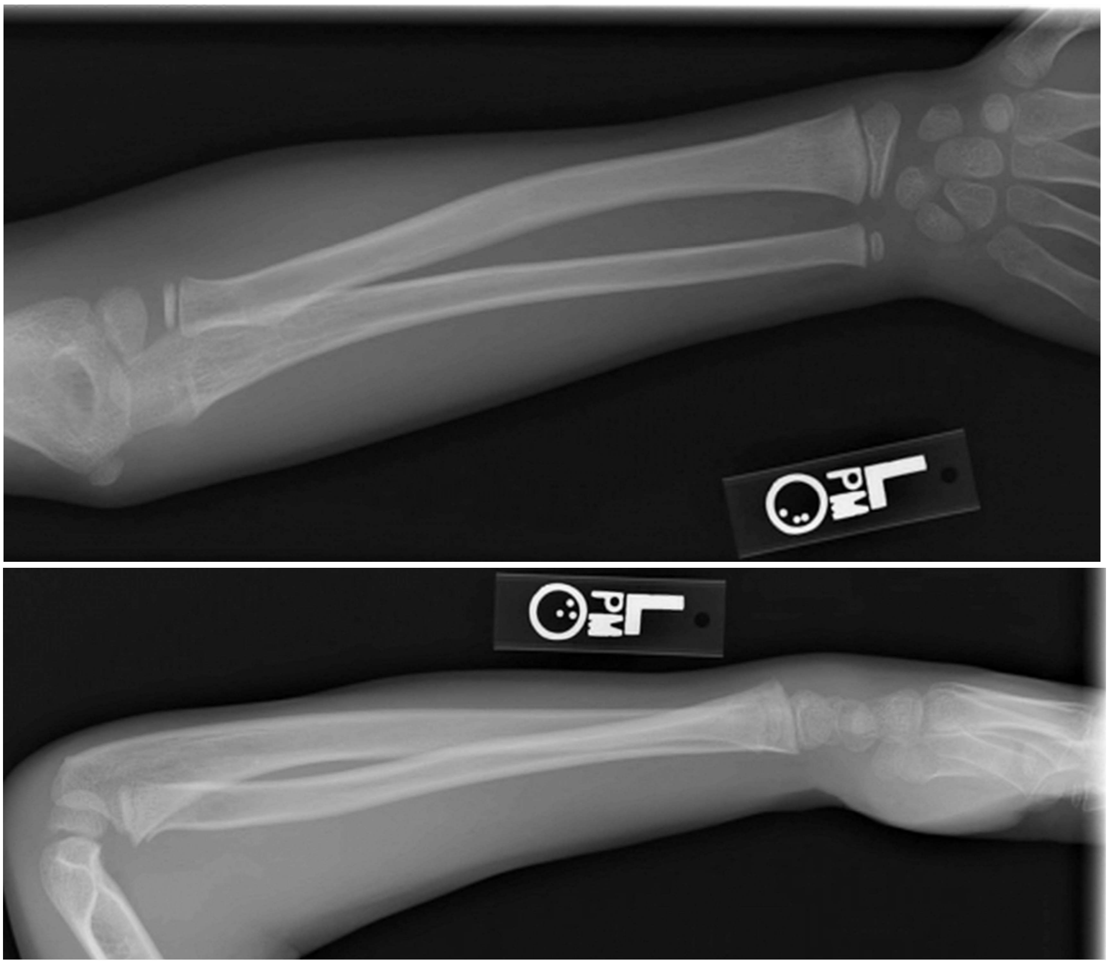

Figure S10 *Fracture gap bridging stage 1: 11-day-old displaced radius fracture (scored as Malone stage 1)*

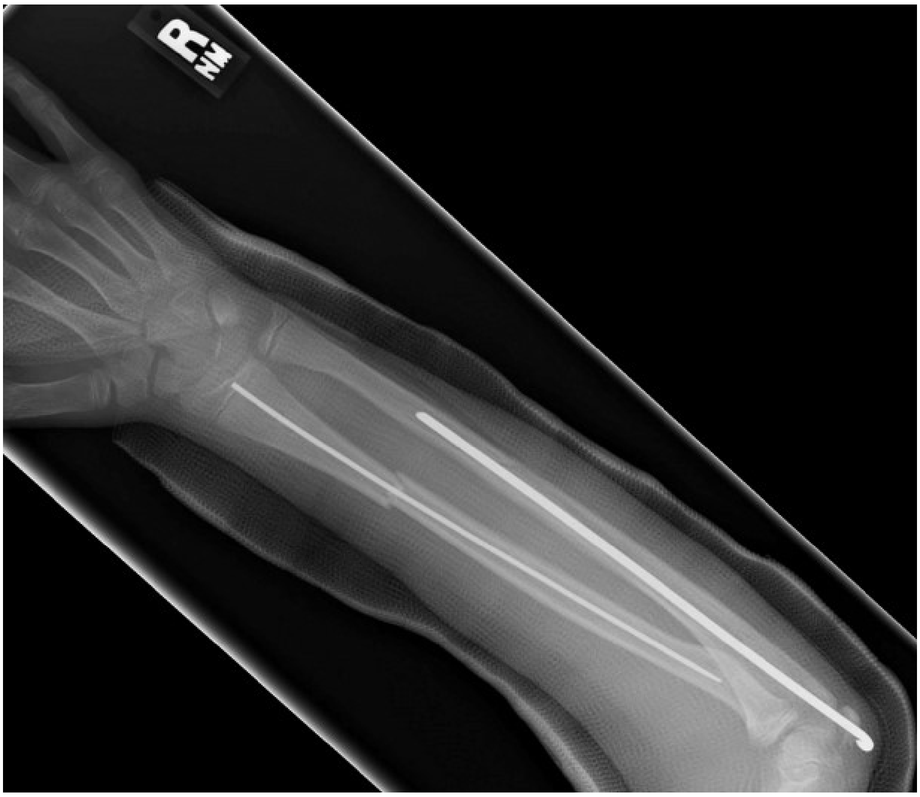

Figure S11 *Fracture gap bridging stage 1: 0-day-old displaced radius and ulna fractures (scored as Malone stage 1)*

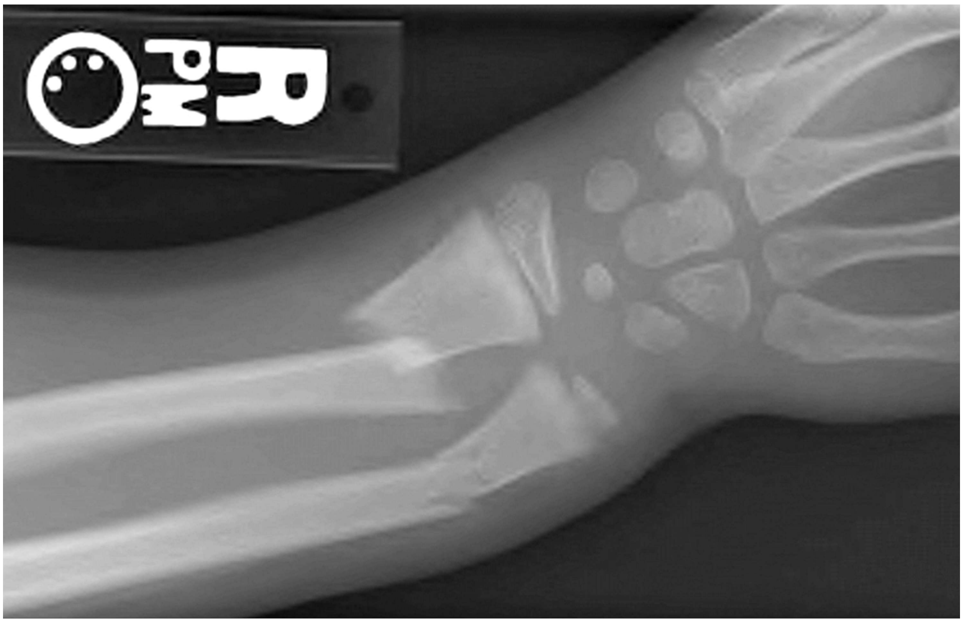

Figure S12 *Fracture gap bridging stage 2: 31-day-old displaced radius and ulna fractures (scored as Malone stage 3)*

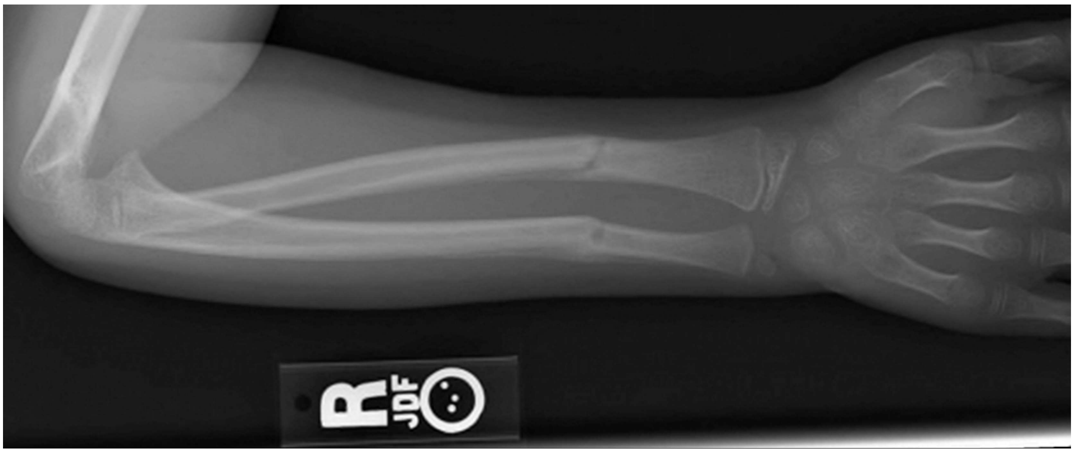

Figure S13 *Fracture gap bridging stage 2: 59-day-old displaced radius and ulna fractures (ulna: scored as Malone stage 4; radius: scored as Malone stage 6)*

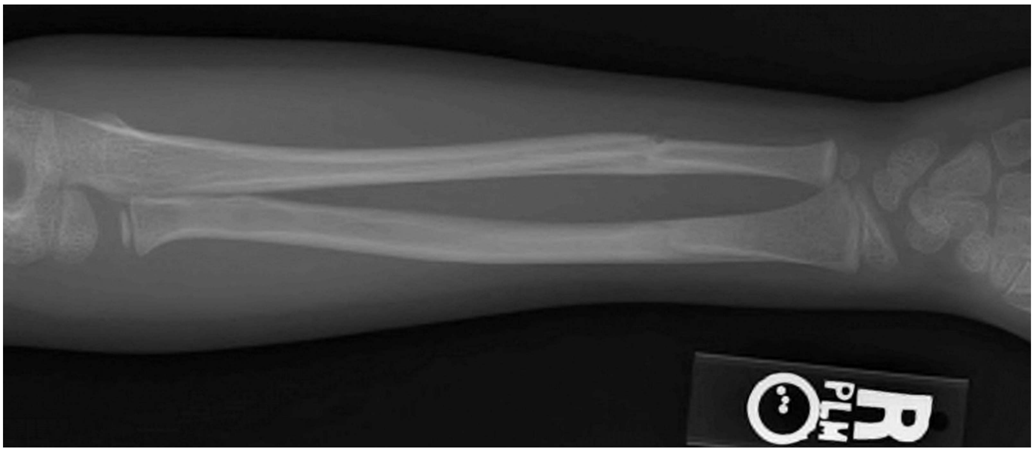
R
PLM

Figure S14 *Fracture gap bridging stage 3: 204-day-old displaced radius and ulna fractures (scored as Malone stage 5)*

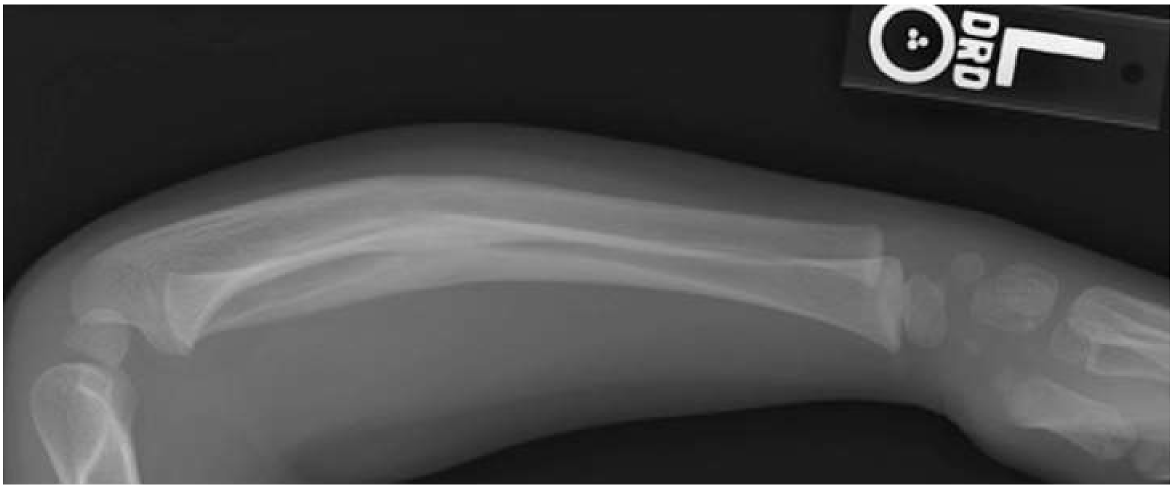

Figure S15 *Sclerosis stage 1: 11-day-old displaced radius and ulna fractures (scored as Malone stage 1)*

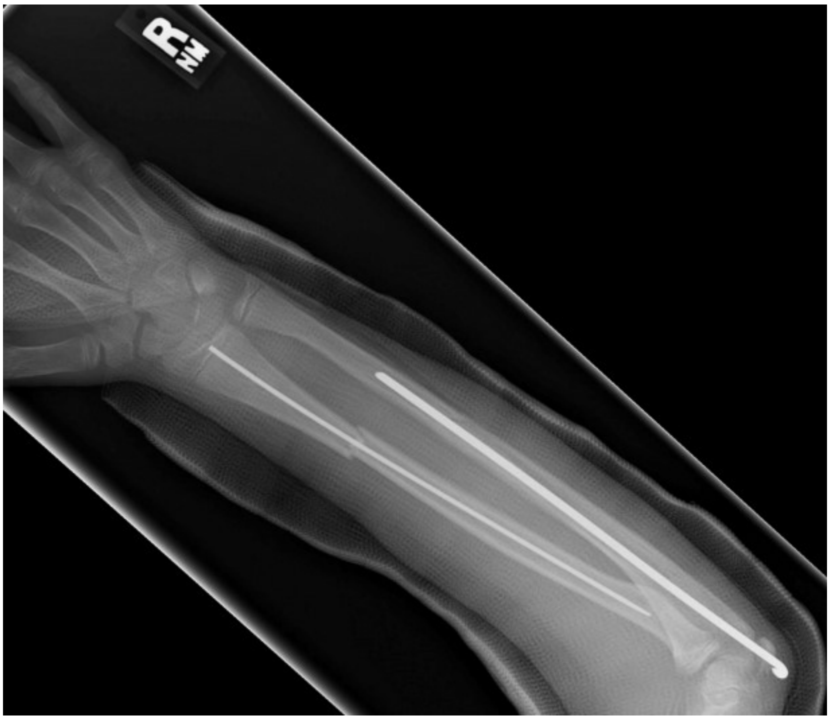

Figure S16 *Sclerosis stage 2: 29-day-old displaced radius fracture (scored as Malone stage 2)*

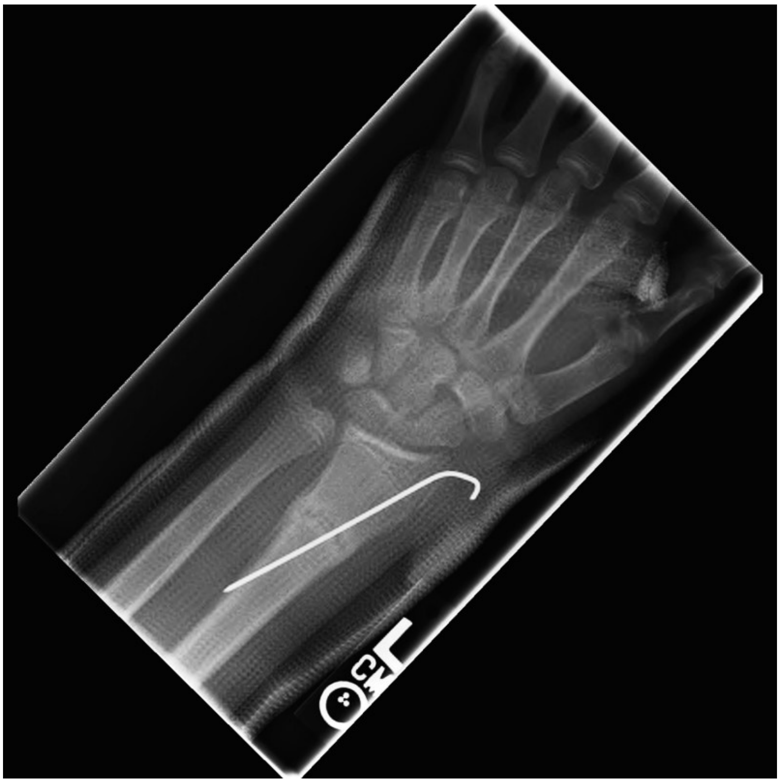

Figure S17 *Sclerosis stage 2: 37-day-old displaced radius fracture (scored as Malone stage 2)*

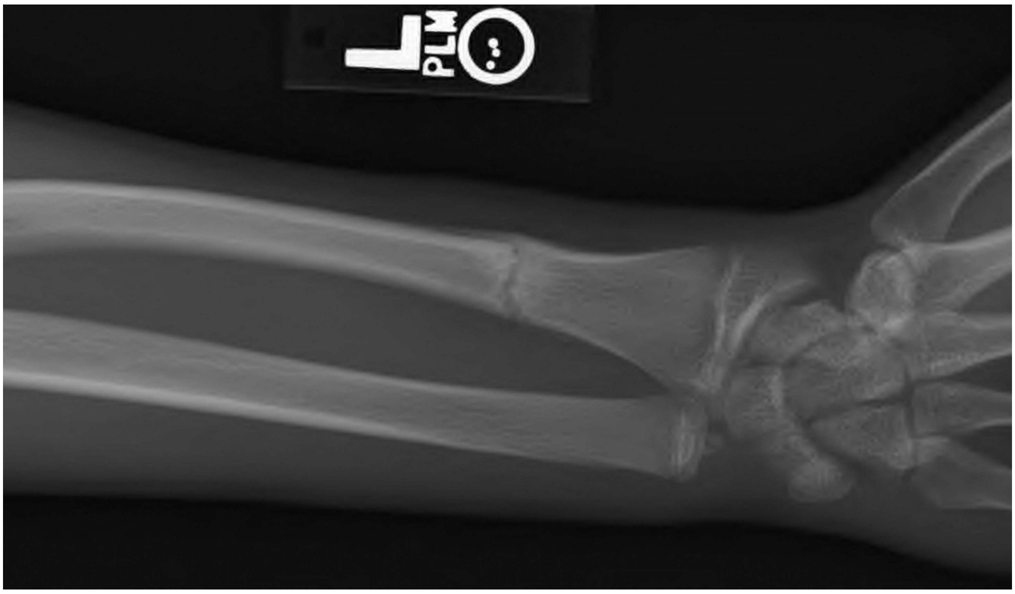

Figure S18 *Sclerosis stage 3: 95-day-old displaced radius fracture (scored as Malone stage 6)*

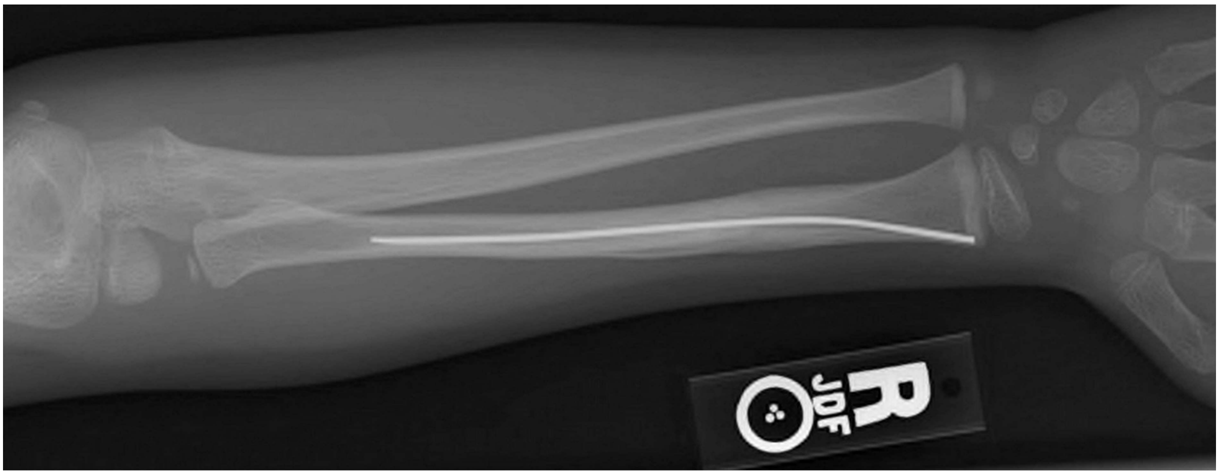

Supplement: Supplementary file 1 [file biology-11-00749-s001.zip › Supplementary figures S1-S18.pdf]
