# Supplementary material for: Postmortem and Antemortem Forensic Assessment of Pediatric Fracture Healing from Radiographs and Machine Learning Classification
Source: Biology (Basel). 2022 May 13;11(5):749. doi: 10.3390/biology11050749 (PMC9138832; doi:10.3390/biology11050749)
Supplement: Supplementary file 1 [file biology-11-00749-s001.zip › supplementary file S1.pdf]

biology MDPI

Supplementary File S1: An R script to implement a random forest model using the Ranger package.

```
library(rsample)     # data splitting
library(ranger)      # Random Forest package
library(readxl)      # Allows reading of Excel files
library(dplyr)       # Data wrangling package
library(tidyverse)

BuckleFracture1 <- read_excel("~/BuckleFracture1.xlsx")
View(BuckleFracture1)

set.seed(123)
buckle_split <- initial_split(BuckleFracture1, prop = 0.7)
buckle_train <- training(buckle_split) #training data for model
buckle_test  <- testing(buckle_split) #test data for final model
View(buckle_train)

# hyperparameter grid search for model
hyper_grid <- expand.grid(
  mtry       = seq(2, 7, by = 1),
  node_size  = seq(1, 31, by = 3),
  num_trees =  seq(50, 500, by=100),
  sampe_size = c(.55, .633, .70, .8),
  OOB_RMSE   = 0
)

# total number of combinations in hypergrid
nrow(hyper_grid)
## [1] 1320

for(i in 1:nrow(hyper_grid))
  {
  # search for optimal model using hypergrid
  model <- ranger(
    formula = FracAge ~ .,
    data = buckle_train,
    num.trees       = hyper_grid$num_trees[i],
    mtry            = hyper_grid$mtry[i],
    min.node.size   = hyper_grid$node_size[i],
    sample.fraction = hyper_grid$sampe_size[i],
    seed            = 123
  )
  # add Out of Bag error to grid
  hyper_grid$OOB_RMSE[i] <- sqrt(model$prediction.error)

}

hyper_grid %>% plyr::arrange(OOB_RMSE) %>% head(12) #Provides parameters for the 12 models with lowest RMSE.
```

```
set.seed(123)
# Optimized Ranger Model
best_ranger<- ranger(
  formula = FracAge ~ .,
  data = buckle_train,
  mtry=4,
  min.node.size = 25,
  num.trees= 250,
  sample.fraction =0.7,
  importance ="impurity")

OOB_RMSE<-vector(mode="numeric", length =100) #Vector to hold RMSE dat

for (i in seq_along(OOB_RMSE))  #Repeated implementation of the optimal model to view variation on OOB RMSE
{
  best_ranger<- ranger(
  formula = FracAge ~ .,
  data = buckle_train,
  mtry=4,
  min.node.size = 25,
  num.trees= 250,
  sample.fraction =0.7,
  importance ="impurity")

  OOB_RMSE[i]<-sqrt(best_ranger$prediction.error)
}
#Histogram of RMSE of OOB sample for 100 implementations of the optimal model
hist(OOB_RMSE, breaks = 10)

best_ranger$variable.importance %>%  #Plot of Relative importance of the predictor variables
  tidy() %>%
  dplyr::arrange(desc(x)) %>%
  dplyr::top_n(8) %>%
  ggplot(aes(reorder(names, x), x)) +
  geom_col() +
  coord_flip() +
  ggtitle("Relative Importance of the Variables")

#The Final Ranger Model
final_ranger<- ranger(
  formula = FracAge ~ .,
  data = buckle_train,
  mtry=4,
  min.node.size = 25,
  num.trees= 250,
  sample.fraction =0.7,
  )
```

```
pred_ranger <- predict(final_ranger, buckle_test)  #Model predictions for test dataset
Predicted<-pred_ranger$predictions #Predicted Fracture Age
Actual<-buckle_test$FracAge #Actual Fracture Age


plot(Actual,Predicted, xlab="Actual Fracture Age", ylab="Predicted Fracture Age", pch=19)
#Scatterplot of Predicted vs. Actual Fracture Age
abline(lm(Predicted ~ Actual), col="red") #Adds regression line

r<-data.frame(Predicted, buckle_test) #Dataframe containing the predicted and actual values
view(r)  #Final dataset
```
