# Supplementary material for: Postmortem and Antemortem Forensic Assessment of Pediatric Fracture Healing from Radiographs and Machine Learning Classification
Source: Biology (Basel). 2022 May 13;11(5):749. doi: 10.3390/biology11050749 (PMC9138832; doi:10.3390/biology11050749)
Supplement: Supplementary file 1 [file biology-11-00749-s001.zip › supplementary file S2.pdf]

biology MDPI

Supplementary File S2: An R script to implement a Gradient Boosting Machine model using the gbm package.

```
library(rsample)     # data splitting
library(gbm)        # basic implementation of gbm model
library(caret)      # an aggregator package for performing many machine learning models
library(pdp)        # model visualization
library(ggplot2)     # model visualization
library(tidyverse)
library(readxl)      #routine for reading excel files
library(xlsx)

setwd("~/")
BFracture <- read_excel("~/BuckleFractures.xlsx")
str(BFracture)

#Coverts character strings to factors
BFracture$logFracAge<-BFracture$logFracAge
BFracture$Age<-BFracture$Age
BFracture$Sex<-factor(BFracture$Sex)
BFracture$Location<-factor(BFracture$Location)
BFracture$Side<-factor(BFracture$Side)
BFracture$Stabilized<- factor(BFracture$Stabilized)
BFracture$Callus<-factor(BFracture$Callus)
BFracture$Sclerosis2<- factor(BFracture$Sclerosis2)
BFracture$Discontinuity2<-factor(BFracture$Discontinuity2)


str(BFracture)
View(BFracture)

set.seed(123)
buckle_split <- initial_split(BFracture, prop = 0.70)  #Creates training and test datasets
buckle_train <- training(buckle_split)
buckle_test  <- testing(buckle_split)
set.seed(123)
write.xlsx(buckle_test,"BuckleTestSet.xlsx", sheetName="Sheet1",
          col.names=TRUE, row.names=TRUE, append=FALSE, showNA=TRUE, password=NULL)#Linear Regression Pre-
dicted vs known age.


# create hyperparameter grid to optimize model
hyper_grid <- expand.grid(
  shrinkage = c(.01, .1, .3),
  interaction.depth = c(1, 3, 5),
  n.minobsinnode = c(5, 10, 15),
  bag.fraction = c(.65, .8, 1),
  optimal_trees = 0,              # a place to dump results
  min_RMSE = 0                    # a place to dump results
)
```

```
# total number of combinations
nrow(hyper_grid)
## [1] 81

# randomize data
random_index <- sample(1:nrow(buckle_train), nrow(buckle_train))
random_buckle_train <- buckle_train[random_index, ]

# grid search
for(i in 1:nrow(hyper_grid)) {

  # reproducibility
  set.seed(123)

  # train model
  gbm.tune <- gbm(
    formula = logFracAge ~ .,
    distribution = "gaussian",
    data = random_buckle_train,
    n.trees = 3000,
    interaction.depth = hyper_grid$interaction.depth[i],
    shrinkage = hyper_grid$shrinkage[i],
    n.minobsinnode = hyper_grid$n.minobsinnode[i],
    bag.fraction = hyper_grid$bag.fraction[i],
    train.fraction = .75,
    n.cores = NULL, # will use all cores by default
    verbose = FALSE
  )

  # add min training error and trees to grid
  hyper_grid$optimal_trees[i] <- which.min(gbm.tune$valid.error)
  hyper_grid$min_RMSE[i] <- sqrt(min(gbm.tune$valid.error))
}

hyper_grid %>%
  dplyr::arrange(min_RMSE) %>%
  head(10)
# plot loss function as a result of n trees added to the ensemble
gbm.perf(gbm.fit, method = "cv")

# for reproducibility
set.seed(123)

# train Final GBM model
gbm.fit.final <- gbm(
  formula = logFracAge ~ .,
  distribution = "gaussian",
  data = buckle_train,
```

```
  n.trees = 100,
  interaction.depth = 3,
  shrinkage = 0.3,
  n.minobsinnode = 5,
  bag.fraction = .65,
  train.fraction = 1,
  n.cores = NULL, # will use all cores by default
  verbose = FALSE
)

#Graph showing relative importance of variables in the final model.
par(mar = c(5, 8, 1, 1))
summary(
  gbm.fit.final,
  cBars = 10,
  method = relative.influence, # also can use permutation.test.gbm
  las = 2
)

# predict values for test data
pred <- predict(gbm.fit.final, n.trees = gbm.fit.final$n.trees, buckle_test)

# results
caret::RMSE(pred, buckle_test$logFracAge)


#Test Set Predictions
Predictions<-data.frame(pred,buckle_test$logFracAge )
view(Predictions)
write.xlsx(Predictions,"GBMOutputBuckle.xlsx", sheetName="Sheet1",
        col.names=TRUE, row.names=TRUE, append=FALSE, showNA=TRUE, password=NULL)#Linear Regression Pre-
dicted vs known age.

# Plots of Log Predicted Fracture aGE VS. Observed Fracture Age
y<-Predictions$pred  #Predicted Ln(fracture_age) values
x<-Predictions$buckle_test.logFracAge  #Actual ln(fracture_age) values

lmFracAge = lm(y~x) #Create the linear regression model of Observed vs. Predicted results
summary(lmFracAge) #Summary of linear regression model

Predicted_Ln_Fracture_Age<-y
Observed_Ln_Fracture_Age<-x
plot(Observed_Ln_Fracture_Age, Predicted_Ln_Fracture_Age) #Scatterplot of Predicted vs Observed Data
abline(lmFracAge) #Regression line laid over scatterplot
```
